# Bidirectional associations between accelerometer-based sleep metrics and mental health symptoms from childhood to late adolescence: data from a Brazilian birth cohort

**DOI:** 10.1186/s12916-025-04296-4

**Published:** 2025-08-15

**Authors:** Marina X. Carpena, Thais Martins-Silva, Andrea Wendt, Inácio Crochemore-Silva, Iná S. Santos, Alicia Matijasevich, Luciana Tovo-Rodrigues

**Affiliations:** 1 Postgraduate Program in Epidemiology, Federal University of Pelotas, Pelotas, Brazil; 2 Human Development and Violence Research Centre (DOVE), Federal University of Pelotas, Pelotas, Brazil; 3 Graduate Program in Health Technology, Pontifícia Universidade Católica Do Paraná, Curitiba, Brazil; 4 Postgraduate Program in Physical Education, Federal University of Pelotas, Pelotas, Brazil; 5 Departamento de Medicina Preventiva, Faculdade de Medicina FMUSP, Universidade de São Paulo, São Paulo, Brazil

**Keywords:** Sleep, Internalizing symptoms, Externalizing symptoms, Adolescence, Childhood, Mental health, Prospective cohort

## Abstract

**Background:**

A bidirectional relationship between sleep and emotional/behavioral problems has been suggested in the literature; however, no study has examined this association longitudinally across multiple developmental stages using objective sleep metrics. This study investigated the reciprocal relationship between sleep and emotional/behavioral symptoms and explored the potential existence of critical or sensitive periods within a Brazilian birth cohort.

**Methods:**

The 2004 Pelotas Birth Cohort Study recruited 4231 children (2196 boys) born in 2004 in Pelotas, Brazil. Emotional/behavioral problems were evaluated using the Strengths and Difficulties Questionnaire (SDQ) at 6, 11, 15, and 18 years of age and analyzed as externalizing and internalizing symptoms. Sleep duration and efficiency were evaluated using actigraphs during the mentioned follow-ups. Cross-lagged panel models were used to assess bidirectionality and critical/sensitive periods.

**Results:**

Higher externalizing symptoms at 6 years predicted longer sleep duration (β = 0.032, *p* = 0.041) and decreased sleep efficiency (β = − 0.034, *p* < 0.022) at age 18. The association was more evident in early adolescence, from 11 to 15 years *β*_sleep duration_ = 0.058, *p* = 0.005; *β*_sleep efficiency_ = − 0.059, *p* < 0.001). A bidirectional relationship was observed for internalizing symptoms and sleep duration from 11 to 18 years (*β*_11-years-sleep duration-15-years-internalizing_ = − 0.039, *p* = 0.008; *β*_15-years-internalizing-18-years-sleep duration_ = 0.061, *p* > 0.001), and higher internalizing scores at age 15 were significantly associated with reduced sleep efficiency at age 18 (*β* = − 0.034, *p* = 0.022).

**Conclusions:**

Higher externalizing symptoms predicted poorer sleep efficiency and longer sleep duration, whereas a bidirectional association with an opposite relationship to internalizing symptoms was found. These results highlight that adolescence is a critical point for both associations.

**Supplementary Information:**

The online version contains supplementary material available at 10.1186/s12916-025-04296-4.

## Background

Sleep health is a multidimensional construct that includes regularity, satisfaction, alertness, appropriate timing, efficiency, and sufficient duration. It plays a vital role in maintaining cognitive functions and mental health, especially in critical developmental periods such as adolescence [1, 2]. Dysfunctional sleep is one of the most common comorbidities among mental health disorders [3–5]. Poor sleep quality, insufficient sleep, and circadian disruption can impair brain function and reduce learning and attention, as well as contribute to the development of mental health conditions, particularly during adolescence [6–9]. 

In recent years, accumulating evidence has suggested a causal relationship between sleep disturbances and mental health disorders, including internalizing conditions like major depressive disorder (MDD) and externalizing disorders such as attention-deficit/hyperactivity disorder (ADHD) and bipolar disorder (BD) [10–12]. Internalizing disorders are generally characterized by emotional symptoms, including anxiety and depression [13], while externalizing disorders are associated with outwardly directed behaviors, such as aggression and hyperactivity [13]. Research suggests that these distinct symptom clusters may have divergent long-term implications. For instance, externalizing behaviors in childhood have been shown to predict adverse outcomes in adolescence, such as arrests and early pregnancies, whereas internalizing symptoms do not exhibit the same predictive value [13, 14].

The relationship between sleep and emotional/behavioral symptoms remains a topic of ongoing debate, with evidence pointing to a complex and bidirectional association. Longitudinal studies consistently indicate that self-reported sleep disturbances, such as reduced sleep duration, may predict and result from externalizing behaviors [15, 16]. Similarly, internalizing symptoms demonstrate bidirectional associations with self-reported sleep behavior, although the evidence for these links is comparatively weaker [17]. While sleep disturbances contribute significantly to the emergence of internalizing difficulties [18, 19], the reverse relationship—where internalizing symptoms precede sleep problems—has not been consistently observed in non-clinical, prospective studies [17, 20].

Despite these findings, the reliance on self-reported measures of sleep introduces potential bias, as objective sleep metrics—widely regarded as more precise and reliable [21, 22]—remain underutilized in this field of research [23]. For instance, using longitudinal data, Kelly et al. demonstrated a bidirectional relationship between objectively measured sleep duration, sleepiness, and externalizing behaviors [23]. Moreover, a recent systematic review underscores the critical need to prioritize the assessment of sleep duration and incorporate objective sleep measures in future studies. It also highlights the pressing need for research involving more diverse and representative populations, as existing evidence is disproportionately derived from European, North American, and Chinese cohorts [16].

Despite the growing body of longitudinal studies investigating the potential direction of the sleep-mental health relationship [17, 20, 24], there are critical gaps in the current literature: (1) there is a lack of evidence from low- and middle-income countries (LMICs), where mental health disorders are highly prevalent [25]; (2) no studies have examined the bidirectional association using accelerometer-based sleep metrics; and (3) prospective studies have primarily been mainly limited to childhood and adolescence. Adopting a developmental perspective that encompasses more than two developmental stages can help identify critical and sensitive periods throughout the life course, providing insights into sustained shifts in health trajectories [26].

This study investigates the reciprocal relationship between sleep and mental health, utilizing accelerometer-based sleep metrics. It also seeks to determine whether there is a specific period during which the impact of sleep duration and efficiency on internalizing and externalizing symptoms (or vice versa) is more pronounced—indicative of a critical or sensitive period—within a Brazilian birth cohort spanning childhood, early, and late adolescence.

## Methods

### Study design and population

This study is based on primary data from the 2004 Pelotas Birth Cohort Study—a prospective, population-based study comprising 99.2% (*n* = 4231; *n* = 2196 boys) of all infants born in 2004 from resident mothers in the urban area of Pelotas (~ 340,000 inhabitants), Southern Brazil. All women with live births were invited to participate. Maternal and child health information was collected within 24 h post-delivery using a structured questionnaire administered by trained interviewers, with newborns examined by a pediatrician. After birth, children underwent assessments at ages 3, 12, 24, and 48 months, as well as at ages 6, 11, 15, and 18 years. In total, 3869 (91.4%), 3799 (89.8%), 3722 (88.0%), and 3565 (84.3%) participants were interviewed at ages 2, 4, 6, and 11 years, respectively. At 15 years old, due to the COVID-19 pandemic, the follow-up rate fell to 48.5% and comprised only a subsample (previously described) [27]. Finally, we interviewed 3489 participants, representing an attrition rate of 85% from the cohort sample at the 18-year follow-up.

Follow-up assessments were conducted at participants’ homes up to 48 months of age and subsequently at 6, 11, and 15 years in a dedicated research clinic operated by the Postgraduate Program in Epidemiology at the Federal University of Pelotas. Comprehensive methodological details have been described elsewhere [27–29]. The 18-year follow-up employed a hybrid approach, integrating household-based interviews with assessments conducted at the research clinic.

### Missing data

Multiple imputation was employed to assign numerical values to sleep and mental health measures among individuals aged 6 to 18, assuming that missing data were missing at random, conditional on the observed data from other variables. To address missingness, we utilized multivariate imputation by chained equations, generating 1000 imputed datasets. Auxiliary perinatal variables incorporated into the imputation model included sex, preterm birth, low birth weight, maternal smoking during pregnancy, self-reported maternal depression, maternal parity, maternal ethnicity, maternal age, maternal education, and family income (details of these variables are provided in the covariates section). Continuous variables were imputed using predictive mean matching. Stata do-files detailing the inclusions and exclusions of the imputation model are available upon request from the corresponding author (MXC). Convergence of all imputation models was confirmed, and Monte Carlo errors for effect estimates were consistently below 10% of their standard errors, indicating that the use of 1000 imputed datasets was adequate [30].

### Emotional and behavioral problems

The Strengths and Difficulties Questionnaire (SDQ) systematically assessed emotional/behavioral problems at all follow-up points. These assessments were conducted through structured interviews with primary caregivers (97% of whom were mothers) by trained psychologists. The interviewers received approximately 40 h of specialized training delivered by an experienced child and adolescent psychologist with expertise in epidemiological assessment, ensuring the data reliability and consistency. In this study, weekly supervision was provided throughout the data collection period. The SDQ has been translated and validated for use in Brazilian Portuguese, ensuring its cultural and linguistic appropriateness for this population [31]. It is a widely used instrument that evaluates emotional and behavioral problems in children and adolescents. It comprises 25 items across five subscales: Emotional Symptoms, Conduct Problems, Hyperactivity/Inattention, Peer Relationship Problems, and Prosocial Behavior. These subscales are categorized into: internalizing symptoms (combining Emotional Symptoms and Peer Relationship Problems), and externalizing symptoms (including Conduct Problems and Hyperactivity/Inattention). The higher the scores, the worse the symptoms.

### Sleep measures

Sleep duration and efficiency were evaluated at the 6-, 11-, 15-, and 18-year follow-ups using ActiGraph accelerometers, model wGT3X-BT (ActiGraph, Pensacola, FL), worn on the non-dominant wrist. Participants were instructed to wear the accelerometer continuously for seven consecutive days, collecting complete data for at least 6 days for the 11 and 15 follow-up assessments. Considering the limited number of accelerometers available at the 6- and 18-year follow-up assessments, specific protocols for data collection were implemented. The instruction was to use the device for 5 to 8 days in the first follow-up and for 5 days in the last [32]. The device tracks sleep and wake periods by recording daily and nightly body movements (or lack thereof). Accelerometers were provided to participants by trained research team members after they visited the research clinic and were later retrieved from participants’ homes on a scheduled date and time. Daily data was downloaded using device-specific software (Actilife) and stored in raw format, with weekly backups conducted. The raw accelerometer data were processed using the GGIR package in R software [33]. This processing involved several critical steps, including autocalibration, non-wear detection, and data cleaning [34, 35]. Autocalibration involved screening for non-movement periods to estimate deviations from the ideal calibration (1 g), with correction factors derived as described in previous research [36]. Non-wear detection was based on standard deviation and value range calculations within 60-min windows, overlapping every 15 min, where at least two of the three axes met predefined thresholds (SD ≤ 13.0 mg and range < 50 mg) [36].

We included participants with at least two valid days of sleep data for ages 6 and 18, and those with at least three valid days of sleep data for ages 11 and 15 were considered to have accurate data. Additionally, automatic sleep detection was performed using the GGIR package [33]. The sleep detection algorithm first identified sleep windows with sustained low acceleration levels. It then searched for 5-min intervals with minimal wrist angle changes (< 3°). These overlapping windows enabled the algorithm to distinguish between sleep periods and wakefulness based on the absence of significant wrist angle [34, 35].

Sleep periods were classified using the algorithm proposed by van Hees et al., which was validated for children and adolescents [37]. Sleep duration was computed by determining the total window time spent asleep, calculated from the overall sleep period (hours). The sleep period was the most prolonged continuous sleep period within a 24-h period (difference between sleep onset and sleep end). On the other hand, sleep efficiency was defined as the percentage of time spent in sleep from bedtime. It is defined as the ratio of total sleep time to bedtime, expressed as a percentage (result = (total sleep time/bedtime) × 100). The sleep efficiency variable was standardized in z-scores for the analyses.

### Covariables

This study used the following maternal sociodemographic and prenatal risk factors from the perinatal study and the 3-month follow-up for sample characterization: age—categorized as < 20, 20–34, and ≥ 35 years; schooling (complete years of formal education)—classified as 0–4, 5–8, and ≥ 9; family income in the month before the delivery (continuous, in Brazilian Real currency); parity (number of previous live or stillbirths—categorized as < 2 and ≥ 2); smoking during pregnancy (categorized as yes and no); and depression during pregnancy (if answered positively to the question: “During pregnancy did you feel depressed or have any nervous symptoms?”). Child characteristics included biological sex (male and female), skin color (white, black, brown, and others), preterm birth (< 37 weeks), and birth weight (measured by hospital staff using 10-g precision pediatric scales, with low birth weight defined as < 2500 g).

### Statistical analysis

The statistical analyses were conducted in STATA v. 18. First, we described the mean rates of sleep measures (sleep duration and sleep efficiency) and externalizing and internalizing SDQ scores in the pre- and post-imputed samples. Finally, we investigated the bidirectional relationship over time between sleep measures and behavioral/emotional problems using four follow-up assessments (at 6, 11, 15, and 18 years) in a cross-lagged panel model (CLPM) analysis. This analysis examines the extent to which mean exposure scores (i.e., sleep measures) at time 1 predict any disease or behavior (i.e., behavioral/emotional problems) at time 2 (a cross-lagged effect), and vice versa. A significant cross-lagged effect often indicates the causality of media exposure at time 1 to behavior at time 2 (and vice versa). The results are expressed in the correlation coefficient (*r*), beta coefficient (*β*), and their 95% confidence interval (95% CI).

### Ethics statement

The 2004 Pelotas Birth Cohort Study received ethical approval from the Medical School Ethics Committee at the Federal University of Pelotas for each follow-up assessment. Mothers were provided with comprehensive information about the study procedures, objectives, voluntary participation, confidentiality rights, and their option to decline participation or withhold specific details. Adolescents provided written assent at the 11-, 15-, and consent at 18-year follow-ups. Cases of severe mental health issues identified by psychologists were assessed and referred to appropriate psychiatric or psychological care facilities as needed.

## Results

### Sample characteristics

Descriptive statistics for the total sample included in the current analysis are presented in Table 1 (*N* = 4.231, 100% of the sample). Among participants, 15% were born preterm (< 37 weeks), 10% were born with low birth weight (< 2500 g), and more than a quarter of the participants (27.5%) had mothers who smoked, had depression symptoms during pregnancy (25.1%), and had ≥ 2 births (60.6%). Regarding ethnicity, 69% of the individuals were classified as white Brazilians. Less than one-third of mothers were younger than 20 years (18.9%), had up to 4 years of education (15.6%), and belonged to the poorest quintile of family income (20.6%) (see Table 1).

**Table 1:** Sample characteristics description among participants of the 2004 Pelotas (Brazil) Birth Cohort

	**Total sample at baseline (*n* = 4231)**	
	***n***	**95% CI**
Preterm birth (< 37 weeks)	612	14.5 (13.5–15.6)
Low birthweight (< 2500 g)	423	10.0 (9.1–11.0)
Maternal smoking during pregnancy	1162	27.5 (26.2–28.8)
Maternal depression	1059	25.1 (23.8–26.4)
Maternal parity (≥ 2)	2563	60.6 (59.1–62.1)
Ethnicity (white)	2726	68.2 (66.7–69.6)
Maternal age (< 20 y)	799	18.9 (17.7–20.1)
Maternal education (0–4 y)	654	15.6 (14.6–16.8)
Family income (poorest)	872	20.6 (19.4–21.9)

### Missing data

The missingness for emotional/behavioral symptoms ranged from 15.8% (*N* = 631) at age 6 to 54.1% (*N* = 2246) at age 15, while missingness for sleep metrics ranged from 21.3% (*N* = 879) at age 11 to 64.9% (*N* = 2692) at age 15. Table 2 describes the sleep measures among the pre- and post-imputation samples. The results show a very similar mean with a slight decrease in standard deviations, indicating improved precision of the measurements. We compared complete cases with missing data for all sleep, mental health, and socioeconomic variables. We found no substantial differences in any key variable across most time points, suggesting that missing data are unlikely to have introduced systematic bias (see Additional Tables S1–S4 for a detailed description). This demonstrates that imputation has no effect or that there are no differences between pre- and post-imputation. The post-imputation sample provides at least 4176 individuals with available data on sleep measures and mental health symptoms.

**Table 2:** Pre- and post-imputation sleep measures and externalizing and internalizing scores description among participants of the 2004 Pelotas (Brazil) Birth Cohort

Measures	Follow-up	Pre-imputation sample		Post-imputation sample	
		***N***	**Mean (SD)**	***N***	**Mean (SD)**
Sleep duration	6	2592	559.59 (54.3)	4200	559.47 (42.9)
	11	3328	482.66 (53.7)	4207	482.70 (48.0)
	15	1484	435.36 (62.1)	4176	435.32 (38.1)
	18	2760	464.52 (93.3)	4203	464.14 (76.2)
Sleep efficiency	6	2590	0.81 (0.06)	4200	0.81 (0.05)
	11	3358	0.85 (0.05)	4207	0.85 (0.04)
	15	1484	0.85 (0.05)	4176	0.85 (0.03)
	18	2697	0.85 (0.06)	4203	0.85 (0.05)
Externalizing	6	3580	4.79 (4.0)	4211	4.80 (3.7)
	11	3563	4.60 (4.2)	4213	4.63 (3.9)
	15	1941	3.99 (3.9)	4187	4.05 (2.7)
	18	3186	4.96 (4.2)	4208	5.02 (3.7)
Internalizing	6	3580	3.62 (3.1)	4211	3.62 (2.8)
	11	3563	4.05 (3.4)	4213	4.07 (3.1)
	15	1941	4.89 (3.7)	4187	4.91 (2.6)
	18	3187	7.38 (4.6)	4208	7.42 (4.1)

### Cross-lagged panel model (CLMP)

**Fig. 1:**
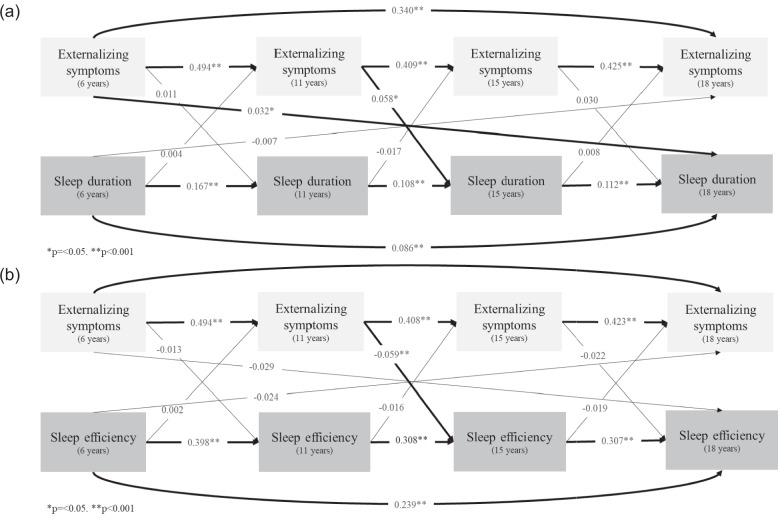
Effect size (beta coefficient) of the bidirectional association tests between sleep duration, sleep efficiency, and externalizing symptoms estimated by cross-lagged panel models among participants in the 2004 Pelotas (Brazil) Birth Cohort. Note: Using data from **a** sleep duration, **b** sleep efficiency, and externalizing score

**Fig. 2:**
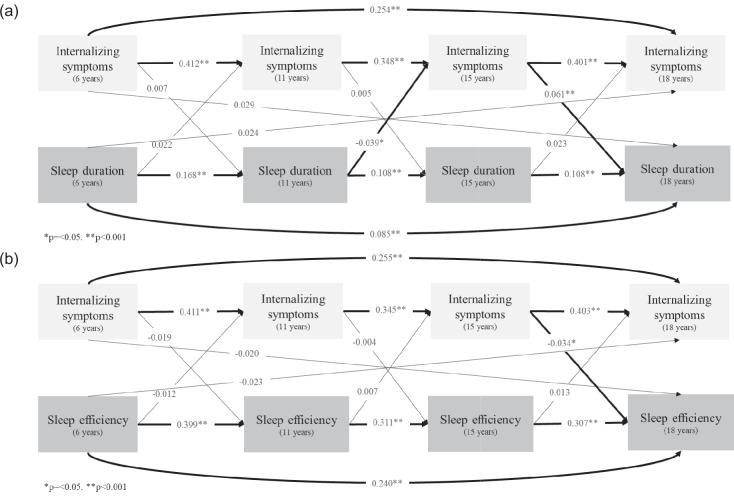
Effect size (beta coefficient) of the bidirectional association tests between sleep duration, sleep efficiency, and internalizing symptoms estimated by cross-lagged panel models among participants in the 2004 Pelotas (Brazil) Birth Cohort. Note: Using data from **a** sleep duration, **b** sleep efficiency, and emotional internalizing score

Sleep measures and both mental health symptoms remained autoregressive associated over 6–18 years (all *p* < 0.001) (see Figs. 1 and 2, Additional files 5 and 6: Tables S5 and S6). Longitudinal results for externalizing SDQ score suggest that higher symptoms predicted a longer sleep duration but worse sleep efficiency, especially during adolescence. Precisely, higher externalizing scores predicted a longer sleep duration at ages 11 to 15 (*β* = 0.058, *p* = 0.005), and from childhood to late adolescence (ages 6 to 18; *β* = 0.032, *p* = 0.041) (see Fig. 1a and Additional file 5: Table S5). Considering sleep efficiency, the longitudinal results indicated that higher externalizing scores at age 11 predicted a slight decrease in sleep efficiency at age 15 (*β* = − 0.059, *p* < 0.001) (see Fig. 1b and Additional file 6: Table S6).

Longitudinal analyses demonstrated a bidirectional relationship between internalizing SDQ scores and sleep duration from ages 11 to 18. Notably, longer sleep duration at age 11 was associated with reduced internalizing scores at age 15 (*β* = − 0.039, *p* = 0.008) (Fig. 2a; Additional file 5: Table S5). Conversely, elevated internalizing scores at age 15 were predictive of longer sleep duration at age 18 (*β* = 0.061, *p* < 0.001) (Fig. 2a; Additional file 5: Table S5). Concerning sleep efficiency, higher internalizing scores at age 15 were significantly associated with reduced sleep efficiency at age 18 (*β* = − 0.034, *p* = 0.022) (Fig. 2b; Additional file 6: Table S6).

## Discussion

This study examined the longitudinal relationship between accelerometer-derived sleep metrics and internalizing and externalizing behaviors across childhood, early adolescence, and late adolescence within a large birth cohort from Brazil. To our knowledge, it represents the first integration of a longitudinal design with accelerometer-based sleep assessments in an LMIC context. Our findings suggest that externalizing behaviors may predict prolonged sleep duration and reduced sleep efficiency, particularly during adolescence (ages 11–15). A bidirectional association was observed between internalizing symptoms and sleep duration from adolescence to late adolescence (ages 11–18). These results highlight the distinct effects of internalizing and externalizing behaviors on sleep.

In this study, we observed that higher externalizing symptoms were prospectively associated with both longer sleep duration and poorer sleep efficiency. These findings are consistent with previous research that links elevated externalizing behaviors and adverse mental health outcomes with suboptimal sleep patterns in young children [38–40]. Importantly, higher externalizing symptoms in our sample predicted decreased sleep efficiency, aligning with patterns observed in studies that utilized self-reported sleep quality measures [17, 20]. Furthermore, studies by Yue et al. [15] and Kelly et al. [23] corroborate the relationship between disrupted sleep and worsening mental health outcomes. A particularly noteworthy study employing objective sleep metrics found that increased variability in weeknight sleep efficiency at age 16 predicted heightened externalizing behaviors at age 17 [23]. Even though the direction was the opposite of ours, these findings underscore the critical need to incorporate objective sleep measures in longitudinal research to elucidate the directionality of these associations more accurately.

The findings regarding sleep duration in this study were particularly compelling. While substantial evidence has consistently shown an association between short sleep duration and poorer mental health outcomes [6, 41, 42], emerging high-quality epidemiological studies suggest that longer sleep duration, particularly when coupled with poor sleep quality, may also be associated with adverse mental health consequences. This underscores the complex and bidirectional relationship between sleep patterns and externalizing behaviors [40]. Specifically, extended sleep durations, often exceeding 8 h, have been correlated with increased inflammation, raising concerns about potential health risks, particularly in older populations [38, 39, 43]. Furthermore, a Mendelian randomization study has indicated that ADHD may contribute to prolonged sleep duration, reinforcing the possibility of a causal relationship between these factors [44]. These observations, in conjunction with our findings, underscore the need for further investigation into the relationship between prolonged sleep duration and mental health outcomes. Future research should broaden its scope to consider short and long sleep durations when exploring their impacts on mental health.

Considering the internalizing scores, our results suggest a complex relationship between sleep and mental health. While longer sleep duration was protective for higher scores of internalizing symptoms, higher internalizing scores predicted shorter sleep duration. Those results partially align with the literature. For example, Zhao et al. [38] confirmed bidirectional relationships between self-reported sleep duration and emotional symptoms, indicating that shorter sleep duration predicts more severe internalizing symptoms and vice versa, particularly during late childhood. On the other hand, we also observed that higher internalizing symptom scores at age 15 predict a longer sleep duration at 18 years, a finding that was not reported by Zhao et al. [38]. The complex bidirectional relationship observed between sleep duration and internalizing symptoms is partially consistent with studies proposing a longitudinal U-shaped pattern [23], where both short and long sleep durations are associated with heightened symptoms. While our data did not support the presence of a U-shaped pattern, these findings collectively underscore the critical importance of addressing sleep hygiene. Both short and prolonged sleep durations are implicated in mental health difficulties, serving as significant comorbid factors related to emotional well-being [45–47]. Finally, our findings did not reveal significant results for sleep efficiency, in contrast to Mulraney et al. [17] and Quach et al. [20], who observed reciprocal effects between sleep quality and emotional or behavioral problems over time.

Unlike most previous studies, our research included late adolescence (18 years), allowing us to demonstrate the bidirectional relationship between sleep and externalizing symptoms from childhood through age 18. This contributes to the developmental perspective literature by highlighting the persistence of these associations over time. Importantly, we observed that adolescence is a sensitive development period for both emotional and behavioral symptoms. We found that higher effects were observed in young people between 11 and 15 years old. Our findings are consistent with previous studies identifying that disruptive sleep or mental health during adolescence might predict current and future difficulties, thus marking adolescence as a critical period for the association between sleep and mental health symptoms [48, 49]. Different critical stages of seeking autonomy mark adolescence [50], developing identity [51], and the brain developmental process [48]. This period may be a time when emotional and behavioral symptoms, as well as sleep-related problems, are more likely to present [48, 50–52]. Additionally, Akbar and collaborators propose that sleep-related problems during early adolescence disrupt brain network dynamics, leading to repetitive negative thoughts and increased vulnerability to internalizing symptoms [53]. Therefore, future research should incorporate additional developmental stages to establish a more comprehensive body of literature that can identify critical periods and advance our understanding of the complex interplay between sleep and mental health symptoms. Also, future studies should directly examine puberty as a developmental mechanism that links sleep, mental health, and other variables to understand better how adolescence represents a critical or sensitive period.

Our results must be interpreted in light of some limitations*.* While our study benefited from actigraphy assessment, the absence of sleep diaries limits our ability to capture subjective sleep-related information and validate the accelerometer-based sleep measures. The use of an automatic sleep detection algorithm also precludes the identification of other critical sleep-related phenotypes, such as sleep latency, and the defined sleep time window does not account for wakefulness within it. Therefore, future studies should investigate these additional sleep metrics to provide a more complete perspective on sleep patterns and their association with mental health. The inconsistent accelerometer wear time across age groups may affect comparability between age groups, and future studies should also consider standardized wear duration to improve reliability and comparability. In addition, we relied solely on maternal reports to assess internalizing and externalizing symptoms, which may be susceptible to reporting bias and might not fully capture the child’s perspective or provide a comprehensive view of symptom severity and context. However, at the 6-year assessment, this was the only feasible source of mental health data. To ensure consistency and avoid introducing bias into the sensitive windows identified in the CLPM, we opted to use the same informant across all waves. Finally, although we used a longitudinal approach, confounding cannot be discarded (review can be assessed [54]). Thus, we highlight the need for further research in this area, particularly using LMIC samples, focusing on adolescent-specific sleep patterns, including other sleep measures when considering the effects of/on mental health, and also understanding other potential moderators in this association.

This study addresses a significant limitation in the existing literature by investigating the bidirectional relationship between sleep and emotional/behavioral symptoms in an LMIC population using accelerometer-derived sleep metrics and longitudinal methodologies. Despite the well-documented impact of sleep on adolescent development, including cognitive function, emotional regulation, and overall well-being [9], and the established role of functional connectivity [52] in predicting sleep duration in youth, data from LMICs have been sparse [9]. Our findings not only elucidate the intricate interplay between sleep and mental health but also explore the presence of critical or sensitive periods in this relationship, thereby contributing to a more comprehensive understanding of adolescent development across diverse general health contexts. 

## Conclusion

In conclusion, our findings highlight that internalizing and externalizing symptoms exhibit distinct sleep associations across the lifespan, with a potential window of opportunity during adolescence. Higher externalizing symptoms are associated with poorer sleep efficiency and longer sleep duration, particularly during adolescence. Conversely, sleep duration exhibits a bidirectional relationship with internalizing symptoms from early adolescence to late adolescence. While our study suggests a critical period during adolescence, a more thorough understanding of the underlying mechanisms and influencing variables is essential. Future studies will be crucial in providing a stronger evidence base for more targeted and effective interventions.

## Supplementary Information

Supplementary material 1. Additional file 1: Table S1 Comparison of complete cases and those with missing information for sleep duration during the follow-ups ages 6, 11, 15, and 18. Additional file 2: Table S2 Comparison of complete cases and those with missing information for sleep efficiency during the follow-ups ages 6, 11, 15, and 18. Additional file 3: Table S3 Comparison of complete cases and those with missing information for externalizing symptoms during the follow-ups ages 6, 11, 15, and 18. Additional file 4: Table S4 Comparison of complete cases and those with missing information for internalizing symptoms during the follow-ups ages 6, 11, 15, and 18. Additional file 5: Table S5 Standardized structural regression coefficients between sleep duration and emotional/behavioral symptoms. Additional file 6: Table S6 Standardized structural regression coefficients between sleep efficiency and emotional/behavioral symptoms.

## Data Availability

The datasets generated and/or analyzed during the current study are not publicly available due to participant confidentiality and data governance policies, but are accessible upon reasonable request. Applications to use the data can be made by contacting the research team of the 2004 Pelotas Birth Cohort Study (see: https://www.epidemio-ufpel.org.br/site/content/faculty/) and submitting the application form available at https://www.epidemio-ufpel.org.br/site/content/studies/formularios.php. A list of administered questionnaires at each wave can be found here: https://www.epidemio-ufpel.org.br/site/content/coorte_2004-en/questionnaires.php. Approved requests will receive a dataset with anonymized participant identifiers and requested variables.

## Abbreviations

ADHD: Attention-deficit/hyperactivity disorder
CI: Confidence interval
CLPM: Cross-lagged panel model
LMICs: Low- and middle-income countries
MDD: Major depressive disorder
SD: Standard deviation
SDQ: Strengths and Difficulties Questionnaire
z-score: Standardized score
